# Pain profiles and variability in temporal summation of pain and conditioned pain modulation in pain‐free individuals and patients with low back pain, osteoarthritis, and fibromyalgia

**DOI:** 10.1002/ejp.4741

**Published:** 2024-10-10

**Authors:** Kristian Kjær‐Staal Petersen, Søren O'Neill, Morten Rune Blichfeldt‐Eckhardt, Casper Nim, Lars Arendt‐Nielsen, Henrik Bjarke Vægter

**Affiliations:** ^1^ Department of Materials and Production, Center for Mathematical Modeling of Knee Osteoarthritis (MathKOA) Aalborg University Aalborg Denmark; ^2^ Department of Health Science and Technology, Faculty of Medicine, Center for Neuroplasticity and Pain Aalborg University Aalborg Denmark; ^3^ Department of Regional Health Research University Hospital of Southern Denmark Odense Denmark; ^4^ Medical Research Unit, Spine Center of Southern Denmark University Hospital of Southern Denmark Middelfart Denmark; ^5^ Department of Anesthesia, Lillebaelt Hospital University Hospital of Southern Denmark Vejle Denmark; ^6^ Department of Sports Science and Clinical Biomechanics Center for Muscle and Joint Health Odense Denmark; ^7^ Department of Gastroenterology & Hepatology, Mech‐Sense, Clinical Institute Aalborg University Hospital Aalborg Denmark; ^8^ Steno Diabetes Center North Denmark, Clinical Institute Aalborg University Hospital Aalborg Denmark; ^9^ Department of Clinical Research, Faculty of Health Sciences University of Southern Denmark Denmark; ^10^ Pain Research Group, Pain Center University Hospital Odense Odense Denmark

## Abstract

**Background:**

Pain profiles (e.g. pro‐ and anti‐nociceptive) can be developed using quantitative sensory testing (QST) but substantial variability exists. This study describes the variability in temporal summation of pain (TSP) and conditioned pain modulation (CPM) in chronic musculoskeletal pain patients, proposes cut‐off values, and explores the association with clinical pain intensity.

**Methods:**

This is a secondary analysis in which TSP and CPM were assessed using cuff algometry in pain‐free subjects (*n* = 69), and patients with chronic low back pain (cLBP, *n* = 267), osteoarthritis (*n* = 134), and fibromyalgia (*n* = 101). Using TSP and CPM from the pain‐free subjects as a reference, four distinct pain profiles TSP (low/high) and CPM (low/high) were created, and differences in clinical pain between pain profiles were explored.

**Results:**

Individual data revealed large inter‐person variability. High TSP and low CPM were found in fibromyalgia (*p* < 0.01) and osteoarthritis (*p* < 0.01) but not cLBP when compared to pain‐free subjects. The proportion of patients classified into the distinct pain profiles was significantly different (*p* < 0.001) with the largest proportion in the high TSP and low CPM group in fibromyalgia (52.5%) and osteoarthritis (41.4%). Clinical pain was not significantly different comparing the pain profiles, and no significant correlations were observed between clinical pain and TSP or CPM.

**Conclusion:**

These results demonstrated substantial inter‐person variability in TSP and CPM in patients with different chronic pain conditions and pain‐free subjects. The proportion of patients with a pro‐nociceptive profile appears larger in fibromyalgia and osteoarthritis, but we found no association to clinical pain.

**Significant Statement:**

This analysis shows that there is variability when assessing TSP and CPM in both pain‐free subjects and patients with chronic pain. A cut‐off for determining when a person is pain‐sensitive is proposed, and data based on this cut‐off approach suggest that significantly more patients with osteoarthritis and fibromyalgia are pain‐sensitive (i.e. higher TSP and lower CPM) compared to pain‐free subjects. This analysis does not find an association between pain sensitivity and clinical pain.

## 1 INTRODUCTION

Chronic pain affects approx. 20% of the world's population (Breivik et al., 2006; Cohen et al., 2021; Goldberg & McGee, 2011; Gureje et al., 2008) with joint‐related and general musculoskeletal (MSK) disorders being the most prevalent diseases (Vos et al., 2016). Chronic MSK pain is complex, and is influenced by a range of different factors (Petersen, 2023).

Different quantitative sensory testing (QST) techniques can be used to quantify the function in the peripheral and central nervous system. Temporal summation of pain (TSP) provides a proxy measure of spinal processes that likely involve wind‐up (Arendt‐Nielsen & Graven‐Nielsen, 2011; Graven‐Nielsen & Arendt‐Nielsen, 2010) and conditioned pain modulation (CPM), which is believed to assess the balance between the descending pain inhibitory and facilitatory systems (Cummins et al., 2020; Nahman‐Averbuch & Timmers, 2023). Recent evidence suggests that TSP and CPM are of interest, as these assessments have been associated with higher risk of chronic postoperative pain (Bossmann et al., 2017; Kurien et al., 2018; Larsen et al., 2021; Petersen et al., 2015, 2018; Rice et al., 2018; Vaegter et al., 2017), poor response to weak analgesics (Arendt‐Nielsen et al., 2016; Edwards et al., 2016; Petersen, Olesen, et al., 2019; Petersen, Simonsen, et al., 2019), and exercise‐based therapies (Hansen et al., 2020; Heredia‐Rizo et al., 2019; Lyng, Andersen, et al., 2022). These observations suggest that the variability in TSP and CPM in patients with chronic MSK pain is important and might be linked to treatment outcomes.

The German Network for Neuropathic Pain has established reference values and the utilization of cut‐off values, which allows for the identification of sub‐groups with different pain profiles (e.g. sensory gain and loss of function) (Rolke et al., 2006; Vollert et al., 2016, 2017). Consensus on such reference values and cut‐off values does not exist for the assessments of TSP and CPM, which are currently holding back the utilization of the assessments in chronic MSK pain research. Some studies have attempted to define cut‐off values for MSK pain using a mean split of patient data (Petersen et al., 2016) or by attempting to use data for pain‐free subjects (Graven‐Nielsen et al., 2015; Vaegter & Graven‐Nielsen, 2016), but none of these cut‐offs have been widely utilized and one explanation for this could be the lack of patient data to support the utility of such cut‐offs.

Using computer‐controlled cuff algometry, a reliable method to assess TSP and CPM (Graven‐Nielsen et al., 2017; Graven‐Nielsen & Arendt‐Nielsen, 2010; Imai et al., 2016; Petersen, Arendt‐Nielsen, et al., 2017; Vaegter et al., 2018; Vaegter & Graven‐Nielsen, 2016), the aims of this study were to (1) describe the inter‐individual variability in TSP and CPM in three patient groups with common chronic pain conditions (chronic low back pain, osteoarthritis and fibromyalgia), (2) use TSP and CPM data from pain‐free subjects to propose cut‐off values in order to create patient sub‐groups with different pain profiles (i.e. pro‐ and anti‐nociceptive profiles), and (3) explore the associations between clinical pain intensity and pro‐and anti‐nociceptive profiles.

## 2 MATERIALS AND METHODS

### 2.1 Subjects

The current study is a secondary analysis of previously published data from patients with chronic low back pain (cLBP) (Holm et al., 2022; O'Neill et al., 2021), knee osteoarthritis (Petersen, Olesen, et al., 2019), fibromyalgia (Blichfeldt‐Eckhardt et al., 2023), and pain‐free subjects (Blichfeldt‐Eckhardt et al., 2023). All studies were granted ethical approval from respective institutional boards and were conducted in accordance with the Helsinki Declaration. All patients and pain‐free subjects provided written informed consent and the protocols for the individual cohorts were approved by the Local Committee on Health Research Ethics (reference numbers: cLBP cohort: S‐20180098, osteoarthritis cohort: N‐20140077, and pain‐free and fibromyalgia cohorts: S‐20160173 and S‐2018003). Participants were recruited across 3 different research sites: (1) The University Hospital Interdisciplinary Pain Center, Odense, Denmark (*n* = 69 pain‐free subjects and *n* = 101 patients with fibromyalgia), (2) The Spine Center of Southern Denmark, Middelfart, Denmark (*n* = 267 patients with cLBP), and (3) Aalborg University Hospital (*n* = 134 patients with knee osteoarthritis). Methods of recruitment for each group are described in detail elsewhere (Blichfeldt‐Eckhardt et al., 2023; Holm et al., 2022; O'Neill et al., 2021; Petersen, Olesen, et al., 2019), but succinctly (1) patients with cLBP as the primary complaint (defined as pain below the lower costal margin and above the inferior gluteal folds, with or without sciatica) were recruited at the Spine Center of Southern Denmark, University Hospital of Southern Denmark, Denmark (Holm et al., 2022; O'Neill et al., 2021); (2) patients with knee osteoarthritis met the American College of Rheumatology criteria for osteoarthritis (Hochberg et al., 2012) and were recruited at Aalborg University Hospital, Denmark (Petersen, Olesen, et al., 2019); (3) individuals with fibromyalgia, diagnosed by a rheumatologist and meeting both the 1990 and the 2016 American College of Rheumatology fibromyalgia diagnosis criteria (Wolfe et al., 2016), were recruited at the University Hospital Odense Interdisciplinary Pain Center, Odense, Denmark (Blichfeldt‐Eckhardt et al., 2023); and (4) pain‐free subjects were recruited via local advertisements in Odense, Denmark.

All Individuals attended a session with assessment of TSP and CPM and individuals with chronic pain conditions also reported clinical pain intensity on a 0‐to‐10 numerical rating scale (NRS) with 0 defined as ‘no pain’ and 10 ‘as worst imaginable pain’.

### 2.2 Assessment of temporal summation of pain and conditioned pain modulation

All studies used computer‐controlled cuff algometry (Cortex Technology, Denmark and Aalborg University, Denmark) to assess TSP and CPM. Test–retest reliability (i.e. the stability of test reliability over time) of computer‐controlled cuff algometry for assessment of pain threshold, TSP, and CPM has been demonstrated in pain‐free subjects (Graven‐Nielsen et al., 2015, 2017; Hviid et al., 2019; Imai et al., 2016; Vaegter et al., 2018) and individuals with chronic pain (Vaegter et al., 2016) to be good‐to‐excellent.

Before the assessment of TSP and CPM, cuff pressure pain threshold (PPT) and cuff pressure pain tolerance (PTT) were assessed (Graven‐Nielsen et al., 2015; Polianskis et al., 2002). Two 13‐cm wide silicone tourniquet cuffs (VBM, Sulz, Germany) were wrapped around the left and right lower legs approximately 8 cm distal from the tibial tuberosity (largest area of the calf muscles). The cuff pressure was increased at one leg at a time with a rate of 1 kPa/s and the maximal pressure limit was 96.5 kPa. Subjects used an electronic 10 cm visual analogue scale (eVAS) to rate the intensity of the pressure‐induced pain in the lower leg in a continuous fashion where zero‐cm and 10‐cm extremes on the VAS were defined as ‘no pain’ and ‘maximal tolerable pain’. The VAS was sampled at 10 Hz and equipped with a stop button. Subjects were informed that they could stop the pressure at any time by pushing the stop button, thus indicating that they were not willing to tolerate more pressure. If the VAS reached a score of 10, the pressure was released from the cuff automatically. The PPT at each leg was defined as the pressure value when the participant rated the sensation of pain as 1 cm on the eVAS. The PTT was defined as the pressure at termination by the stop button or when the eVAS rating reached 10 cm.

### 2.3 Assessment of temporal summation of pain

To assess cuff‐induced TSP, 10 repeated pressure stimulations (1 s duration and 1 s interval between stimuli) were delivered to the left leg with an intensity equivalent to the PTT recorded during the previous assessment. Pressure with an intensity equivalent to the PTT was chosen to ensure that the first stimulation was perceived as somewhat painful although not extremely painful because of the short stimulation time. Subjects rated their pressure pain intensity on the eVAS continuously during the sequential stimulations without returning the eVAS to zero between the 10 stimulations. The eVAS score immediately after each stimulus was extracted. TSP‐effect was calculated as the difference between the mean pain intensity rating of the 8th to 10th stimuli minus the mean pain intensity of the 1st to 3rd stimuli. A TSP‐effect above 0 reflects an increase in pain ratings during repeated stimulations, while a TSP‐effect below 0 reflects a decrease in pain ratings.

### 2.4 Assessment of conditioned pain modulation

To assess cuff‐induced CPM, the conditioning stimulus was delivered by the tourniquet cuff wrapped around the right lower leg (conditioning stimulus cuff). Within 1 s, the cuff was inflated to a pressure equal moderate pain (either VAS = 5 or 70% of PTT), and the pressure was kept constant throughout the CPM protocol (maximum of 96.5 s). Pressure intensity equal to VAS = 5 or 70% of PTT was chosen to ensure that the conditioning cuff was above PPT and thus would be perceived as moderately painful, as recommended (Yarnitsky et al., 2015). Five seconds after inflation of the conditioning stimulus cuff, the cuff on the left leg (test stimulus cuff) was inflated at a rate of 1 kPa/s, and the PPT and PTT were reassessed as described above. Subjects were informed that they should focus their attention on the test stimulus cuff. The CPM‐effect was defined as the difference in PPT with and without the conditioning stimulus (i.e. PPT recorded during conditioning stimulation minus PPT recorded at baseline), with positive scores indicating an inhibitory effect of the conditioning stimulus on the test stimulus.

### 2.5 Statistical analyses

Categorical variables were expressed in absolute figures and proportions of the total. Continuous data were reported as means and SDs. *p* values < 0.05 were considered significant. Statistical analyses were run in SPSS Statistics (version 28; IBM, Armonk, NY).

### 2.6 Investigating group differences and inter‐personal variability in TSP and CPM

Prior to defining groups, the difference in cuff‐induced TSP‐ and CPM‐effects between pain‐free subjects and the three chronic pain groups was compared with 1‐way ANOVA. Additionally, Individual TSP and CPM data from pain‐free subjects and the chronic pain groups were displayed in scatterplots to investigate inter‐personal variability. To support the scatterplots, the range of TSP and CPM data is presented for each cohort and the coefficient of variation (CV) was calculated.

### 2.7 Developing a cutoff value for TSP and CPM

Four distinct groups of pain patients were created based on TSP and CPM, using TSP (low/high) and CPM (low/high) as categorical variables. According to the literature, a pain‐sensitive person could be explained by a high TSP and a low CPM value (Yarnitsky, 2010).

Since there is no consensus on cut‐off criteria for TSP and CPM, we chose a cut‐off for high TSP and low CPM being the mean value for TSP and CPM in the healthy population including the upper or lower limit of uncertainty around the mean estimates. We chose the upper and lower 95% limits around the mean estimates expecting that the ‘true’ estimates would fall below or above this cutoff 95% of the time if we would run the assessments in the pain‐free population again. Thus, subjects were classified as having high TSP if the TSP‐effect was greater than the upper bound of the 95% CI of the mean of the TSP value in pain‐free subjects and low TSP if the TSP‐effect was equal to or less than the upper bound of 95% CI of the mean of the TSP value in pain‐free subjects. Subjects were classified as having low CPM if the CPM‐effect was smaller than the lower bound of the 95% CI of the mean CPM value in pain‐free subjects and high CPM if the CPM‐effect was equal to or larger than the lower bound of the 95% CI of the mean CPM value in pain‐free subjects. This classification was chosen to acknowledge that some otherwise pain‐free subjects will be pain‐sensitive when assessed using a TSP and CPM paradigm (Hertel, Ciampi de Andrade, et al., 2024; Izumi et al., 2022) and when applying this method, this allowed for pain‐free subjects to be classified as pain‐sensitive to both the TSP and CPM paradigm.

Using this classification, four groups were developed for each group (i.e. pain‐free subjects, low back pain, osteoarthritis, and fibromyalgia) categorized by (1) low TSP and high CPM, (2) high TSP and high CPM, (3) low TSP and low CPM, and (4) high TSP and low CPM.

### 2.8 Investigation of clinical pain and TSP and CPM

The proportion of subjects categorized into each of the four‐pain profiles was analysed by chi‐squared tests. Clinical pain intensity scores between the four distinct TSP and CPM groups were analysed with 1‐way ANOVA for fibromyalgia, cLBP, and knee osteoarthritis, separately, and associations between pain intensity and TSP and CPM were explored with Pearson coefficients. In case of significant factors, Bonferroni pairwise comparisons correcting for multiple comparisons were used. Effect size for difference in pain intensity between pain profiles was reported as Cohen's *d*.

## 3 RESULTS

### 3.1 Subject characteristics

The combined sample (*n* = 571) was 58% female (*n* = 330) with an average (SD) age of 51 (15) years. Sex, age, and clinical pain intensity scores across groups are presented in Table 1.

**TABLE 1:** Demographics, clinical pain (assessed using a numerical rating scale, NRS), cuff pressure pain threshold (PPT) pressure tolerance threshold (PTT), temporal summation of pain (TSP), and conditioned pain modulation (CPM). Data are presented as means (and standard deviations). The coefficient of variation (CV) was reported for all measures.

	Pain‐free (*n* = 69)	Low back pain (*n* = 267)	Knee OA (*n* = 134)	Fibromyalgia (*n* = 101)
Female (%)	43 (62.3%)	117 (43.8%)	71 (53.0%)	99 (98%)
Age (years)	37.7 (16.2)	50.2 (13.4)	64.1 (9.4)	47.3 (10.7)
Clinical pain intensity (NRS: 0–10)	‐	7.1 (2.3)	6.7 (2.3)	7.3 (1.7)
PPT (kPa)	25.2 (12.0)	22.6 (10.4)	20.3 (10.9)	15.1 (7.1)
PTT (kPa)	59.1 (22.1)	53.5 (20.4)	38.8 (16.6)	34.9 (18.5)
TSP (cm)	1.18 (1.26)	1.44 (1.49)	2.05 (1.78)	2.16 (1.91)
CPM (kPa)	8.75 (10.7)	6.42 (11.5)	1.19 (8.9)	0.89 (6.39)
CV TSP (%)	106.78	103.47	86.83	88.43
CV CPM (%)	122.29	179.13	172.27	717.98

### 3.2 Studying group differences and inter‐personal variability in TSP and CPM

**FIGURE 1:**
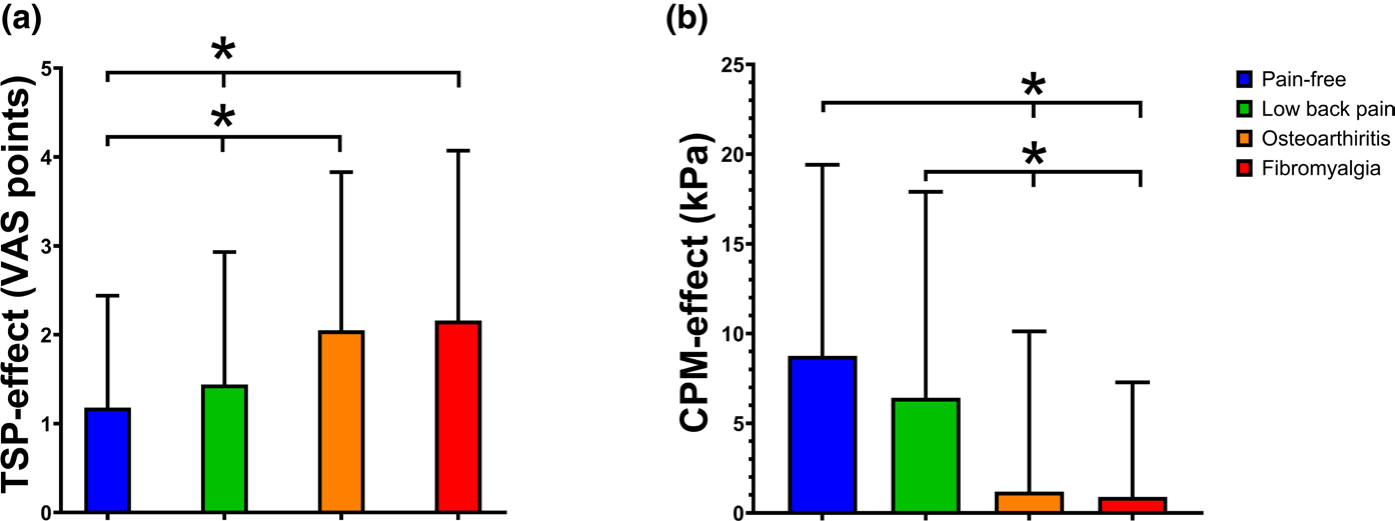
Mean (±SD) (a) temporal summation of pain effect (TSP‐effect) and (b) conditioned pain modulation effect (CPM‐effect) from pain‐free subjects (blue); subjects with chronic low back pain (green), knee osteoarthritis (orange), and fibromyalgia (red). * Indicates significant difference (*p* < 0.05) when compared to pain free‐subjects and subjects with low back pain.

**FIGURE 2:**
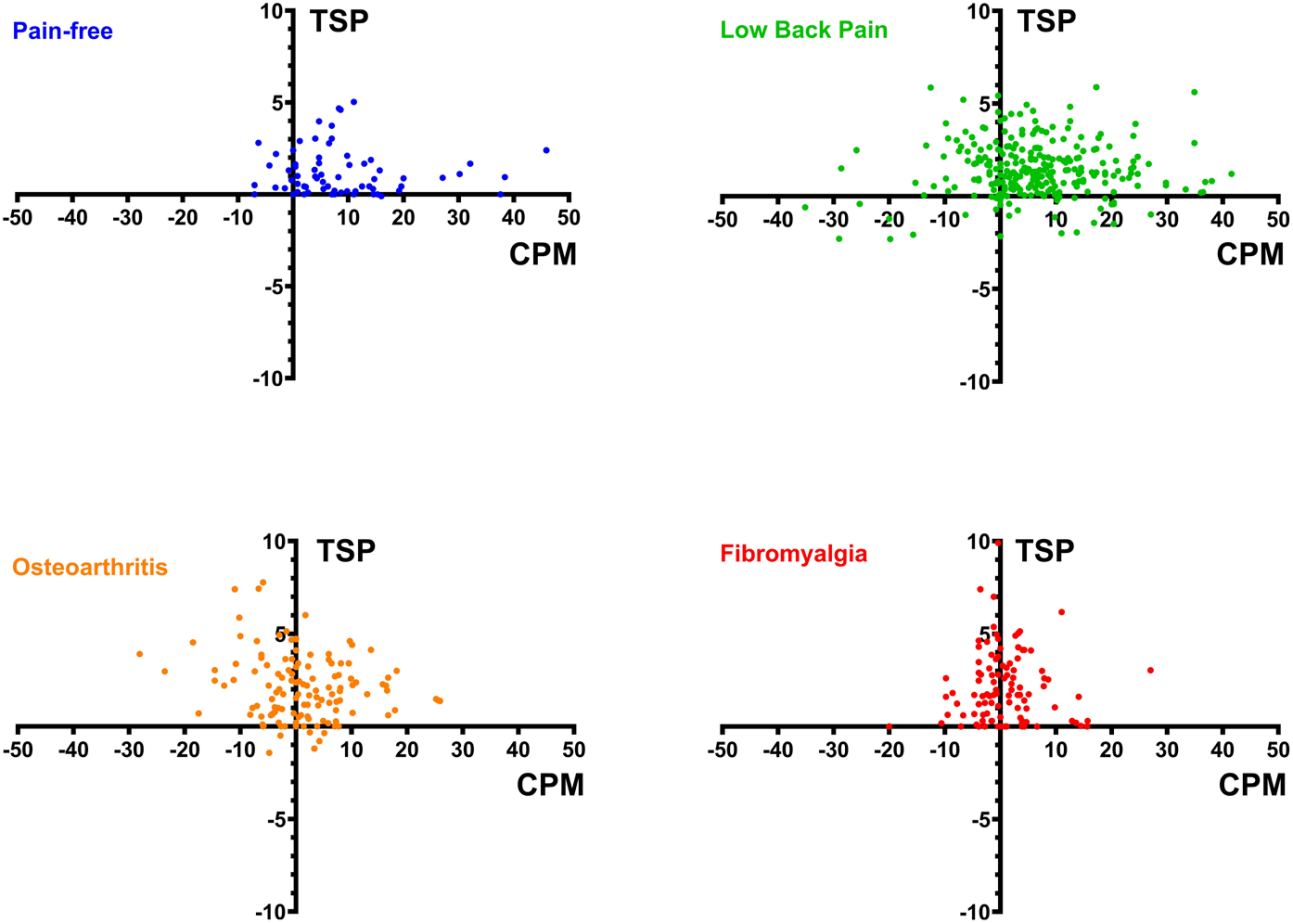
Individual temporal summation of pain effect (TSP, *y*‐axis, cm) and conditioned pain modulation effect (CPM, *x*‐axis, kPa) from pain‐free subjects (blue, *n* = 69); patients with chronic low back pain (green, *n* = 267), knee osteoarthritis (orange, *n* = 134), and fibromyalgia (red, *n* = 101).

The ANOVA demonstrated a significant group difference when comparing TSP‐effect from the four cohorts (*F*_3,568_ = 9.49, *p* < 0.001). Post hoc test showed that both pain‐free subjects and patients with cLBP had lower TSP‐effect than both patients with fibromyalgia and knee osteoarthritis (*p* < 0.003, Figure 1a). The ANOVA demonstrated a significant group difference in the CPM‐effects (*F*_3,564_ = 16.19, *p* < 0.001). Post hoc test showed that both pain‐free subjects and patients with cLBP had higher CPM‐effect than patients with both fibromyalgia and knee osteoarthritis (*p* < 0.001, Figure 1b). Individual TSP‐ and CPM‐effects for all subjects are illustrated in Figure 2. The range for TSP‐ and CPM‐effects for the groups were: pain‐free subjects: TSP = [−0.1; 5.0], CPM = [−7.0; 45.9], cLBP: TSP = [−2.3; 5.9], osteoarthritis: TSP = [−1.4; 7.8], CPM = [−28.1; 25.9], and fibromyalgia: TSP = [0.0; 9.9], CPM = [−20.0; 27], and the CV for all assessments were more than 80%, which suggests substantial inter‐personal variability in all of the four cohorts.

### 3.3 Defining a cut‐off value for temporal summation of pain and conditioned pain modulation

**FIGURE 3:**
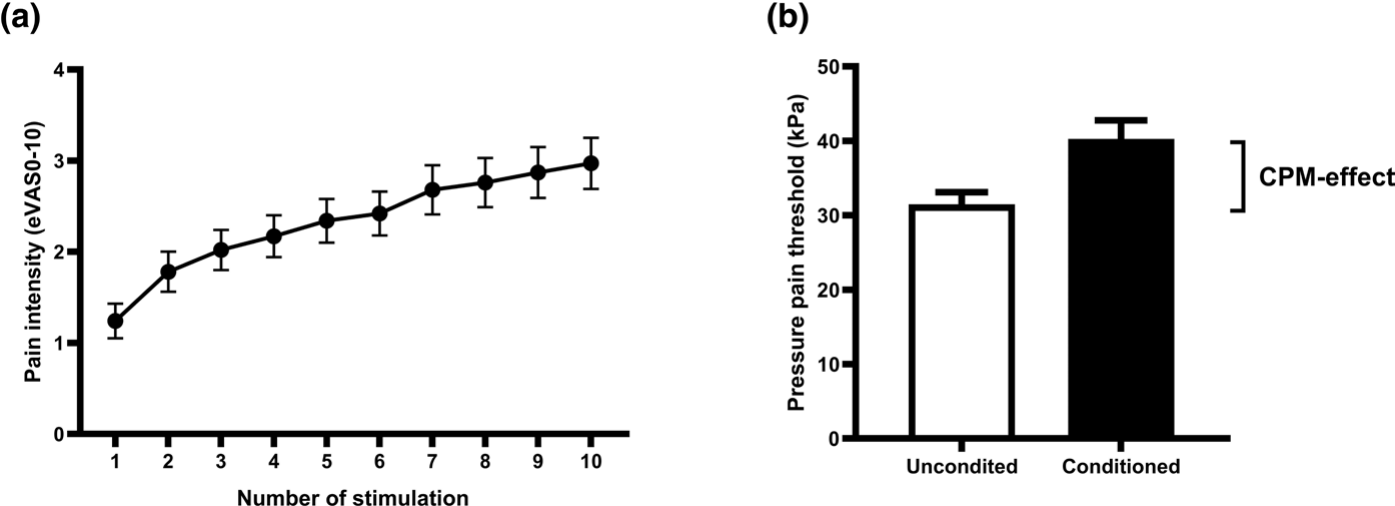
Mean (±SEM) scores for (a) pain ratings for 10 identical painful stimuli utilized to assess temporal summation of pain and (b) pressure pain thresholds assessed without (white) and with (black) painful conditioned stimulus utilized to assess conditioned pain modulation from pain‐free individuals.

The VAS scores immediately after the 10 repeated cuff stimulations for pain‐free subjects are illustrated in Figure 3a. The mean TSP‐effect in pain‐free subjects was 1.18 (95% CI: 0.88–1.49; Table 1).

The PPT scores with and without conditioning for the pain‐free subjects are illustrated in Figure 3b. The mean CPM effect in pain‐free subjects was 8.75 kPa (95% CI: 6.15–11.35; Table 1).

Based on these results from healthy pain‐free subjects, a cut‐off value was defined using the upper and lower bounds of the 95% confidence intervals, and as such a TSP‐effect above 1.49 VAS points was considered high and a CPM‐effect below 6.15 kPa was considered low.

### 3.4 Subgroups based on CPM and TSP pain profiles

**FIGURE 4:**
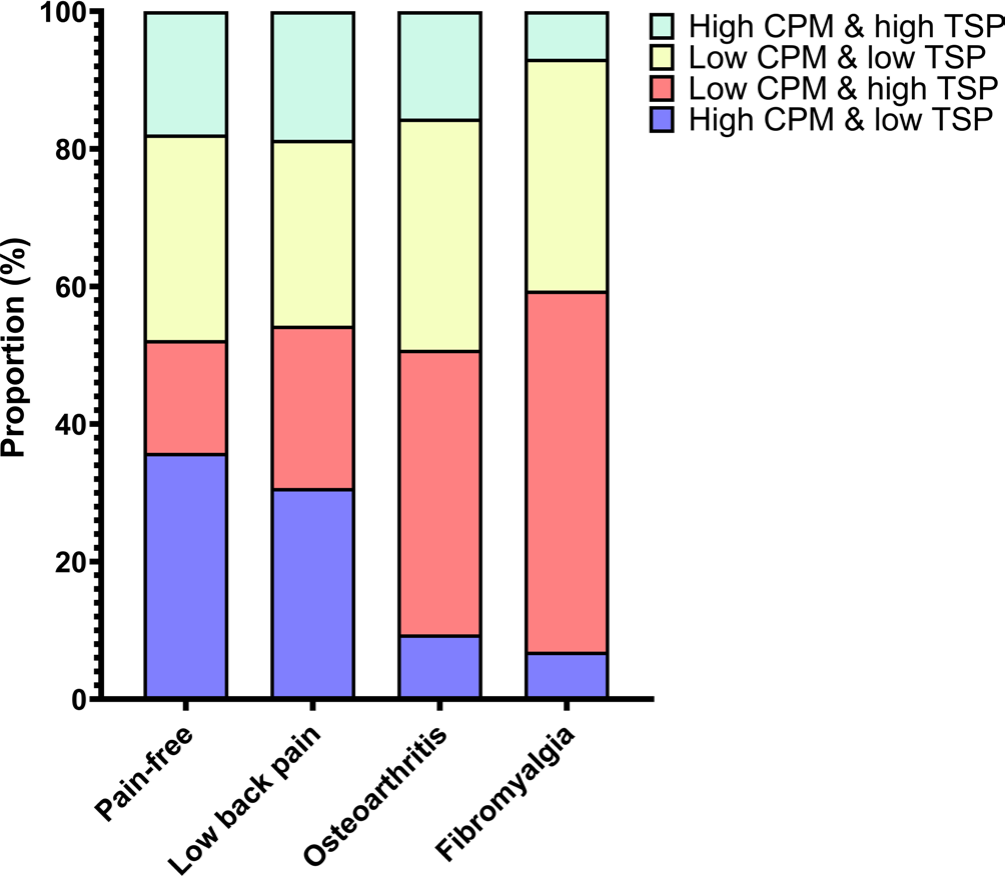
Proportion of pain‐free subjects and patients with chronic low back pain, knee osteoarthritis, and fibromyalgia with different pain profiles based on temporal summation of pain (TSP) and conditioned pain modulation (CPM).

The proportion of subjects classified into the four different TSP and CPM pain profiles were significantly different between groups and are shown in Figure 4 (*X* ^(9)^ = 70.66, *p* < 0.001). The proportion of subjects with low TSP and high CPM (anti‐nociceptive pain profile) was highest in pain‐free subjects (35.8%) and lowest in patients with fibromyalgia (6.9%) followed by patients with knee osteoarthritis (9.4%). The proportion of subjects with high TSP and low CPM (pro‐nociceptive pain profile) was highest in fibromyalgia (52.5%) followed by knee osteoarthritis (41.4%) and lowest in cLBP (23.6%) and pain‐free subjects (16.4%).

### 3.5 CPM and TSP pain profiles and their association with clinical pain intensity

Clinical pain intensity scores are reported in Table 2 for the different pain profiles for patients with cLBP, knee osteoarthritis, and fibromyalgia. Across the three patient groups, the ANOVAs showed no significant differences in clinical pain intensity between the four pain profile groups (*p* > 0.22). The correlation of clinical pain intensity with TSP and CPM was not significant for fibromyalgia (TSP: *r* = 0.15, *p* = 0.14; CPM: *r* = 0.04, *p* = 0.70), knee osteoarthritis (TSP: *r* = 0.14, *p* = 0.13; CPM: *r* = −0.15, *p* = 0.11), and cLBP (TSP: *r* = 0.02; *p* = 0.81; CPM: *r* = 0.14, *p* = 0.14). In patients with FM (Cohen's *d*: 0.61) and knee OA (Cohen's *d*: 0.67) pain intensity scores were numerically higher in the pro‐nociceptive pain profile group than in the anti‐nociceptive pain profile group; however, statistical comparison was not performed due to few participants in the anti‐nociceptive groups.

**TABLE 2:** Clinical pain intensity scores (mean ± SD) measured using a numerical rating scale (0–10, ‘0’ indicating no pain and ‘10’ indicating worst imaginable pain) from patients with chronic low back pain, osteoarthritis, and fibromyalgia and with different pain profiles.

	Normal CPM and normal TSP	Impaired CPM & facilitated TSP	Impaired CPM and normal TSP	Normal CPM and facilitated TSP
Low back pain	7.22 (2.25)	6.53 (2.84)	7.1 (2.84)	7.91 (2.49)
Osteoarthritis	5.75 (2.14)	7.1 (2.18)	6.64 (2.44)	6.42 (2.22)
Fibromyalgia	6.86 (1.57)	7.5 (1.58)	6.82 (1.98)	7.86 (1.22)

## 4 DISCUSSION

This exploratory study showed significantly higher TSP and significantly lower CPM in patients with fibromyalgia and knee osteoarthritis when compared to pain‐free subjects and that substantial inter‐person variability in TSP and CPM exists among pain‐free subjects and patients with chronic low back pain, knee osteoarthritis, and fibromyalgia. The distributions of TSP and CPM data from pain‐free participants were used to dichotomize patients' TSP and CPM responses as either high or low. The pro‐nociceptive pain profile (high TSP and low CPM) was more prevalent in patients with fibromyalgia and knee osteoarthritis compared with chronic low back pain. Finally, the results suggest no association between clinical pain intensity in any of the pain groups and TSP or CPM.

### 4.1 Variability in temporal summation and pain and conditioned pain modulation

The variability in TSP and CPM can arise from several factors, which interfere with the assessments. TSP and CPM have been criticized for poor reliability (Kennedy et al., 2016, 2020) but it is current unclear if these reliability issues are due to technologically issues or as recent data might suggests, that this variability might originate from biological variability (i.e. biological factors that interfere with the assessments). The assessment of TSP and CPM using cuff algometry has consistently shown good‐to‐excellent reliability (Graven‐Nielsen et al., 2015, 2017; Hviid et al., 2019; Imai et al., 2016; Vaegter et al., 2018; Vaegter & Graven‐Nielsen, 2016). Several larger studies on patients with chronic MSK pain and pain‐free subjects have demonstrated large variability in the individual TSP and CPM responses (Izumi et al., 2022; Larsen et al., 2024; Petersen et al., 2016; Vaegter & Graven‐Nielsen, 2016). The current study aligns with these previous publications: whilst the current study finds considerable overlaps between groups due to other and unknown sources of between‐subject variation. Several biological factors can influence the variability of TSP and CPM assessments and studies have demonstrated that sleep deprivation can impact both TSP and CPM (Campbell et al., 2015; Hertel et al., 2023; Staffe et al., 2019), mental factors (Christensen et al., 2020; Meints et al., 2019), and inflammation (Giordano et al., 2024; Schaible, 2004). Future studies should investigate some of the underlying reasons for why some people are more pain‐sensitive than others even within the same pain condition.

### 4.2 Comparing findings from patients with chronic pain and pain‐free subjects

Systematic reviews and meta‐analyses tend to conclude that TSP is facilitated and CPM is impaired in patients with osteoarthritis and fibromyalgia when compared to pain‐free subjects (Arendt‐Nielsen et al., 2018; Arendt‐Nielsen, Skou, et al., 2015; Lyng, Thorsen, et al., 2022; McPhee et al., 2020) and the current study supports such a conclusion. The data from larger studies on chronic LBP have been inconsistent in demonstrating significant differences in TSP and CPM when comparing patients with chronic LBP and pain‐free subjects (Corrêa et al., 2016; France et al., 2016; Gerhardt et al., 2016; O'Neill et al., 2014; Tesarz et al., 2015, 2016). A 2020 systematic review and meta‐analysis of more than 1500 patients with LBP and more than 500 pain‐free subjects did find significant differences comparing TSP and CPM but the effect sizes were small (McPhee et al., 2020). The current study did not demonstrate significant differences in TSP and CPM in patients with chronic LBP compared to pain‐free individuals. The patients recruited for the current analysis included a wide range of different problems and clinical presentations, from non‐specific low back pain to surgical conditions. This heterogeneity in clinical presentation is likely to have diluted any systematic effect of the clinical condition of LBP on pain sensitivity in our results.

### 4.3 Clinical pain and quantitative sensory testing

Previous studies have demonstrated associations between clinical pain and QST findings in patients with chronic pain (Arendt‐Nielsen et al., 2010; Arendt‐Nielsen, Egsgaard, et al., 2015). In patients with neuropathic pain, PPTs have been correlated to self‐reported deep pain, but thermal qualities of ongoing pain are not related to thermal hyperalgesia (Gierthmühlen et al., 2019). Also, sensory phenotyping of patients with neuropathies has not been able to identify specific subsets of patients with and without neuropathic pain (Blesneac et al., 2018; Forstenpointner et al., 2021; Held et al., 2019; Themistocleous et al., 2016). Within MSK pain, the evidence is contradictory, and some studies have used sensory phenotyping and found differences in clinical pain (Petersen et al., 2015; Vaegter & Graven‐Nielsen, 2016) whereas others have found none (Petersen et al., 2016). A recent study attempted to use QST, psychological factors, and quality of life to explain pain in osteoarthritis and demonstrated that QST could significantly explain some variability of pain in osteoarthritis but that the explanatory value was low and lower than both pain catastrophizing and quality of life (Hertel, Arendt‐Nielsen, et al., 2024), suggesting that QST might have a limited explanatory value for MSK pain. The current study is the largest study to investigate pain profiles in patients with MSK to date and did not report significant differences within the different pain profiles and did not report significant association between clinical pain intensity and TSP or CPM, suggesting that TSP and CPM are not associated to clinical pain in patients with cLBP, osteoarthritis, and fibromyalgia. The current analysis is based on specific cut‐off values, inspired by the cut‐off values utilized by the German Network for Neuropathic Pain (Baron et al., 2017; Vollert et al., 2017), and future studies should further investigate if different cut‐off values should be investigated.

Recent data, mainly from patients with MSK pain, suggests that QST might hold predictive value for chronic pain after surgery (Izumi et al., 2017; Kurien et al., 2018; Larsen et al., 2021; Petersen et al., 2015, 2023), and these findings suggest that QST might be useful in identifying ‘pain‐vulnerable’ patients who are at risk of a poor outcome following surgical procedures. This hypothesis is supported by recent data from studies of human experimental pain models, where higher pain sensitivity in otherwise pain‐free subjects was demonstrated to be associated with more severe pain following delayed onset muscle soreness pain models when compared to less pain‐sensitive subjects (Kristensen et al., 2021). Conclusively, these data could suggest that QST findings are not associated with chronic MSK pain, but that QST might be utilized to identify subjects at risk of pain in the future.

### 4.4 Limitations

The primary focus of the current analysis was to investigate differences in TSP and CPM profiles in pain‐free subjects and in patients with chronic LBP, knee osteoarthritis, and fibromyalgia. The current analysis was not powered to investigate differences in clinical pain intensity scores between different pain profiles within the different pain cohorts and this should be considered when reading this work.

The current study is based on secondary data from three different centres, and this could introduce variability into the data as different assessors were involved in the data collection, which should be considered. The current study utilized the computer‐controlled cuff algometer, which is an automated assessment tool, and the assessor only needs to mount cuffs on the lower and instruct the participants on the procedures. All the procedures and the instructions for the participants were standardized. In addition, the cuff algometers have demonstrated good‐to‐excellent reliability (Graven‐Nielsen et al., 2017; Imai et al., 2016; Petersen, Vaegter, & Arendt‐Nielsen, 2017; Vaegter et al., 2018) and is as reliable or more reliable as other available tools.

Due to the exploratory nature of this analysis, we were unable to control for age in the different groups. Age and sex have been found to impact QST findings (mainly pain thresholds) (Edwards et al., 2003; Mogil, 2012; Riley et al., 1998) but the impact on TSP and CPM is conflicting (Mertens et al., 2021; Petersen, Vaegter, & et al., 2017; Popescu et al., 2010; Skovbjerg et al., 2017), why one could argue that a potential effect might be small. The four cohorts presented in the current manuscript were not recruited to have similar sex and age distribution and this should be considered when interpreting the results.

Recent data suggest that TSP and CPM can be influenced by a range of factors, such as psychological factors (Christensen et al., 2020), poor quality of sleep (Hertel et al., 2023; Staffe et al., 2019), and potentially inflammation (Schaible, 2014), and a range of other factors and these factors as well as comorbidities (i.e. competing pain conditions) are not accounted for in the current analysis.

The current study utilized a healthy pain‐free group of 69 people to develop the TSP and CPM cut‐off, but the TSP and CPM profiles are not described in another pain‐free group, which would increase the validity. Future studies should test the cut‐off criteria in another pain‐free group to explore the validity of the criteria from the current study.

## 5 CONCLUSION

The current study is the first large‐scale analysis of how to utilize TSP and CPM to investigate distinct pain profiles and identify substantial inter‐person variability in pain‐free subjects and patients with chronic low back pain, knee osteoarthritis, and fibromyalgia. The study found higher TSP and lower CPM in patients with knee osteoarthritis and fibromyalgia, but not in patients with chronic low back pain when compared to pain‐free subjects. Based on a cut‐off value defined from pain‐free subjects, the proportion of subjects with high TSP and low CPM was highest in fibromyalgia (52.5%) and knee osteoarthritis (41.4%) and lowest in chronic low back pain (23.6%) and pain‐free subjects (16.4%). The current study was unable to link the pro‐ and anti‐nociceptive profiles to clinical pain or to demonstrate significant correlations between clinical pain and TSP and CPM. Future studies should investigate the underlying factors for the inter‐person variability in the TSP and CPM.

## FUNDING INFORMATION

The Center for Neuroplasticity and Pain (CNAP) is supported by the Danish National Research Foundation (DNRF121) and the Center for Mathematical Modelling of Knee Osteoarthritis (MathKOA) is funded by the Novo Nordisk Foundation (NNF21OC0065373). The Innovation Fund Denmark (j.no. 136‐2014‐5); The Aalborg University Talent Management Programme (j.no. 771126); The Shionogi Science Program; the TaNeDS Europe grant; The Danish Rheumatism Association (Nos. R155‐A4866‐B1363, R175‐A6088‐B1363); Aase og Ejnar Danielsens Foundation; Karen S Jensens Foundation; Oberstinde Kirsten Jensa la Cours legat; Professor, Overlæge Sophus H. Johansens Foundation of August 23, 1981; Foundation of the Danish Association for Anaesthesia and Intensive Care; Fonden til Lægevidenskabens Fremme; the Development Foundation, Lillebaelt Hospital; OUH Fund for Free Research; Research fund of the Region of Southern Denmark; Læge Sofus Carl Emils og Hustru Olga Doris Friis grant; and Danish Foundation for the Advancement of Chiropractic Research and Post Graduate Education are kindly acknowledged for the possibility to collect the clinical data.

## CONFLICT OF INTEREST STATEMENT

The authors declare no conflicts of interest.
